# Elevated IKKα Accelerates the Differentiation of Human Neuronal Progenitor Cells and Induces MeCP2-Dependent BDNF Expression

**DOI:** 10.1371/journal.pone.0041794

**Published:** 2012-07-27

**Authors:** Ali Khoshnan, Paul H. Patterson

**Affiliations:** Biology Division 216-76, California Institute of Technology, Pasadena, California, United States of America; University of Insubria, Italy

## Abstract

The IκB kinase α (IKKα) is implicated in the differentiation of epithelial and immune cells. We examined whether IKKα also plays a role in the differentiation and maturation of embryonic human neuronal progenitor cells (NPCs). We find that expression of an extra copy of IKKα (IKKα+) blocks self-renewal and accelerates the differentiation of NPCs. This coincides with reduced expression of the Repressor Element Silencing Transcription Factor/Neuron-Restrictive Silencing Factor (REST/NRSF), which is a prominent inhibitor of neurogenesis, and subsequent induction of the pro-differentiation non-coding RNA, miR-124a. However, the effects of IKKα on REST/NRSF and miR-124a expression are likely to be indirect. IKKα+ neurons display extensive neurite outgrowth and accumulate protein markers of neuronal maturation such as SCG10/stathmin-2, postsynaptic density 95 (PSD95), syntaxin, and methyl-CpG binding protein 2 (MeCP2). Interestingly, IKKα associates with MeCP2 in the nuclei of human neurons and can phosphorylate MeCP2 *in vitro*. Using chromatin immunoprecipitation assays, we find that IKKα is recruited to the exon-IV brain-derived neurotrophic factor (BDNF) promoter, which is a well-characterized target of MeCP2 activity. Moreover, IKKα induces the transcription of BDNF and knockdown expression of MeCP2 interferes with this event. These studies highlight a role for IKKα in accelerating the differentiation of human NPCs and identify IKKα as a potential regulator of MeCP2 function and BDNF expression.

## Introduction

IKKα is a component of the IKK complex (α, β, γ), which is an important regulator of NF-κB pathway and plays a major role in cell proliferation/differentiation and inflammation. IKKα also regulates the production of active p52 NF-κB factor, which is essential for the development of the immune system [1]. Moreover, IKKα has anti-inflammatory properties and can inhibit the IKKβ/NF-κB activity and lower the expression of inflammatory cytokines [2], [3]. Recent studies have identified several NF-κB-independent functions for IKKα [4]. IKKα localizes to the nucleus and phosphorylates proteins such as CREB binding protein (CBP), the silencing mediator of retinoid and thyroid hormone receptor (SMRT), forkhead box A2 (FOXA2), and β-catenin. All of these proteins are expressed in the brain and are implicated in various aspects of neurodevelopment [4]–[9]. In animal models, IKKα-mediated phosphorylation of histone-3 (H3) and CBP contributes to memory reconsolidation in the hippocampus [10]. Moreover, IKKα phosphorylates the estrogen receptor and promotes estrogen-regulated gene expression [11]. Estrogen is a neurosteroid that modulates dendritic growth and synaptogenesis in the central nervous system [12]. Thus, IKKα may play a role in neurodevelopment.

IKKα is constitutively active in human neuronal progenitor cells (NPCs). Moreover, the level and activity of IKKα decreases in neurons exposed to DNA damaging agents whereas elevation of IKKα is neuroprotective and augments neuronal resiliency to stress [13]. Here, we report that expression of an extra copy of IKKα accelerates the differentiation and maturation of human embryonic NPCs. Our data also identify IKKα as a modifier of MeCP2, which is a prominent regulator of neuronal gene expression [14]. Thus, manipulating the levels and activity of IKKα may be a useful strategy to enhance neuronal differentiation and regulate MeCP2 activity.

## Results

### Elevation of IKKα affects the proliferation and differentiation of human neuronal progenitor cells

**Figure 1:**
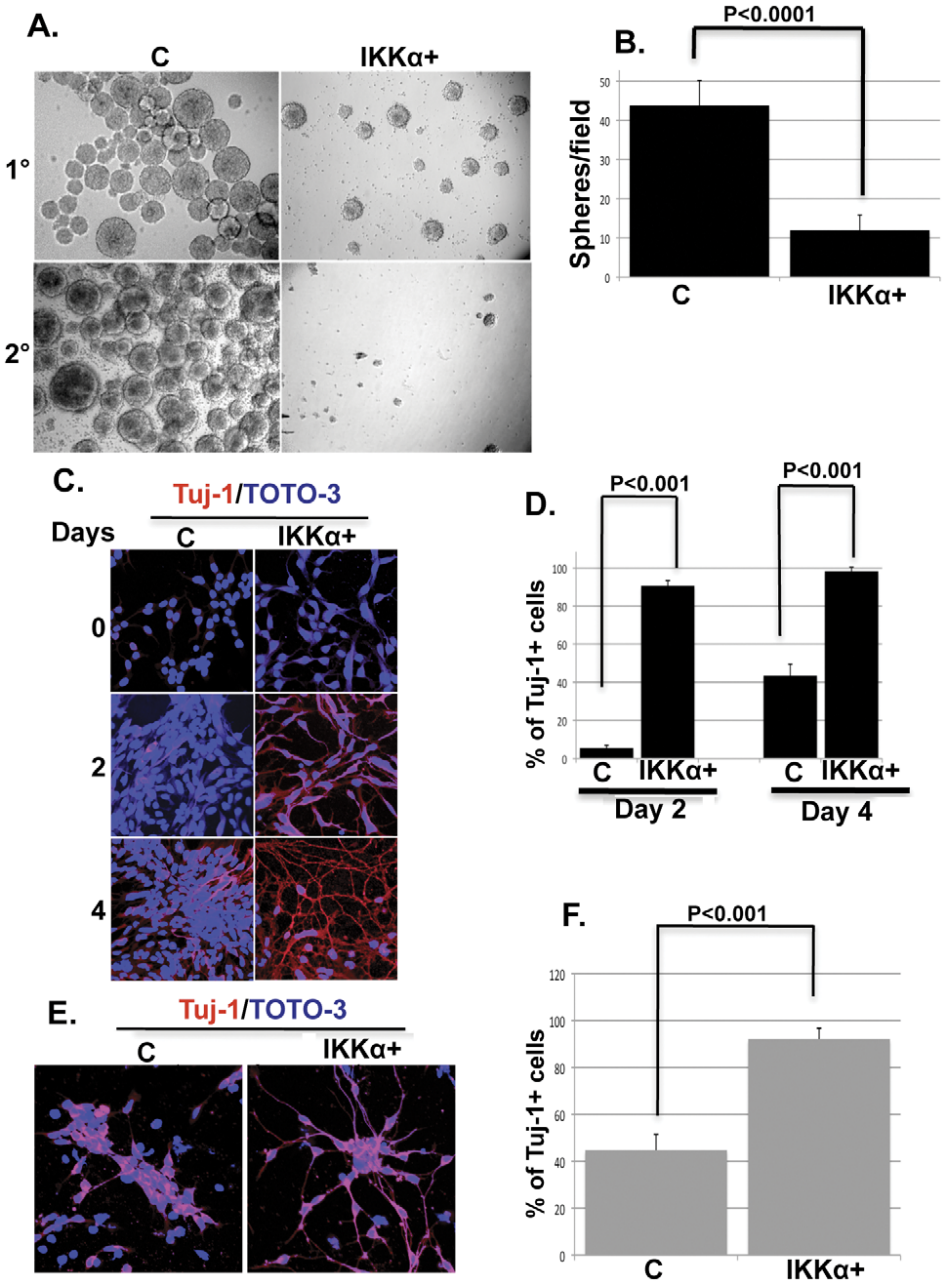
Effects of IKKα on the proliferation of MESC2.10 NPCs. (**A**) Elevated IKKα impairs neurosphere formation of MESC2.10 NPCs. The neurosphere assay was carried out as described in Methods. Representative micrographs of primary (1°) (top panels) and secondary (2°) neurospheres (bottom panels) formed by control (C) and IKKα+ NPCs are shown. Assays were done in triplicate. (**B**) Quantification of neurospheres reveals a significant deficit in the IKKα+ NPCs compared to controls. Six culture wells were counted in each condition and averaged. P value was obtained using student's t-test. (**C**) Elevated IKKα induces the differentiation of MESC2.10 cells when v-myc expression is repressed. Control and IKKα+ NPCs were cultivated on laminin in the absence (time 0) or the presence of doxycycline for 2 or 4 days. Cells were stained for Tuj-1 expression. Representative micrographs obtained with a confocal microscope are shown. The DNA stain TOTO-3 was used to identify nuclei. (**D**) The % of Tuj-1 positive cells in day 2 and day 4 cultures is shown. (**E**) IKKα+ neurospheres undergo spontaneous neuronal differentiation. Day 6 dissociated neurospheres were plated on laminin and stained for Tuj-1 after 24 h. Representative micrographs obtained with a confocal microscope are shown. (**F**) The % of Tuj-1 positive in day 2 and day 4 cells is shown. For D and F, total and Tuj-1 positive cells were counted in 6 different confocal images and the % positive was calculated. P values were obtained using student's t-test.

IKKα regulates the differentiation of several cell types including epithelial and immune cells including monocytes, B cells, and regulatory T cells [15]–[19]. Interestingly, the level of IKKα protein is increased several fold during monocyte-to-macrophage differentiation [18]. The focus of this study was to determine whether elevation of IKKα alters the proliferation and/or the differentiation of an embryonic human mesecephalic NPC line (MESC2.10). Unlimited proliferation of MESC2.10 cells is regulated by a tetracycline-regulated (tet-off) v-myc and the addition of mitogenic factor, basic fibroblast growth factor-2 (bFGF-2). Upon shutting down the expression of v-myc by doxycycline and removal of FGF-2, MESC2.10 NPCs can differentiate into neurons expressing dopaminergic markers [20]. Expressing an extra copy of IKKα in MESC2.10 cells (IKKα+) (Fig. S1) has no visible effect on proliferation when v-myc is expressed (data not shown). Employing a neurosphere assay, which is used to study the self-renewal of neuronal stem cells (NSCs) [21], we find that MESC2.10 cells proliferate for several generations in the absence of v-myc. While IKKα+ cells also form neurospheres, they are smaller in size and the numbers are significantly reduced (Fig. 1A top panels and B). To extend these findings, primary neurospheres were dissociated into single cell suspensions and cultured in a second round in the presence of FGF-2 and doxycycline. Although control NPCs form secondary neurospheres, this property is completely lost in IKKα+ NPCs (Fig. 1A. bottom panels). Thus, elevated IKKα interferes with the self-renewal of MESC2.10 NPCs.

To examine whether the reduced proliferation of IKKα+ progenitors is due to precocious differentiation, we cultivated cells on a laminin substrate in proliferating medium (+bFGF-2) with the addition of doxycycline to repress v-myc expression, which blocks neurosphere formation of the IKKα+ but not of the control NPCs (Fig. 1A). Staining cells for the neuronal differentiation marker β-tubulin III (Tuj-1), we do not find any Tuj-1-positive cells in either control or IKKα+ NPCs when cells express v-myc (Fig. 1C top panels). However, the majority of IKKα+ NPCs express Tuj-1 by the 2^nd^ day after the addition of doxycycline. This is in contrast to control NPCs, which continue to proliferate under these conditions and ∼5% of the cells stain positively for Tuj-1 by the 2^nd^ and ∼45% by the 4^th^ days (Fig. 1C, D). By the 4^th^ day, IKKα+ NPCs develop extensive neurite outgrowth (Fig. 1C lower right panel), which is a hallmark of neuronal differentiation *in vitro* [22]. We also plated day 6 dissociated neurospheres (Fig. 1A) on laminin and examined for Tuj-1 after 24 h of further cultivation in the presence of bFGF-2 and doxycycline. More than 95% of the IKKα+ NPCs express Tuj-1 and develop prominent neurite outgrowth. Under these conditions, ∼50% of the control cells also stain positively for Tuj-1 but have no detectable neurite outgrowth (Fig. 1E, F). Control and IKKα+ NPCs express Nestin, which is a marker of proliferating NPCs (Fig. S2A). However, growth conditions that promote the differentiation of IKKα+ NPCs (Fig. 1C, E), do not significantly affect the level of Nestin. Nestin accumulates in the neurites of dissociated day 6 IKKα+ neurospheres whereas it is predominantly in the cytoplasm of control cells (Fig. S2B). It is possible that the turnover and/or reduction of Nestin expression requires a longer cultivation of IKKα+ NPCs.

**Figure 2:**
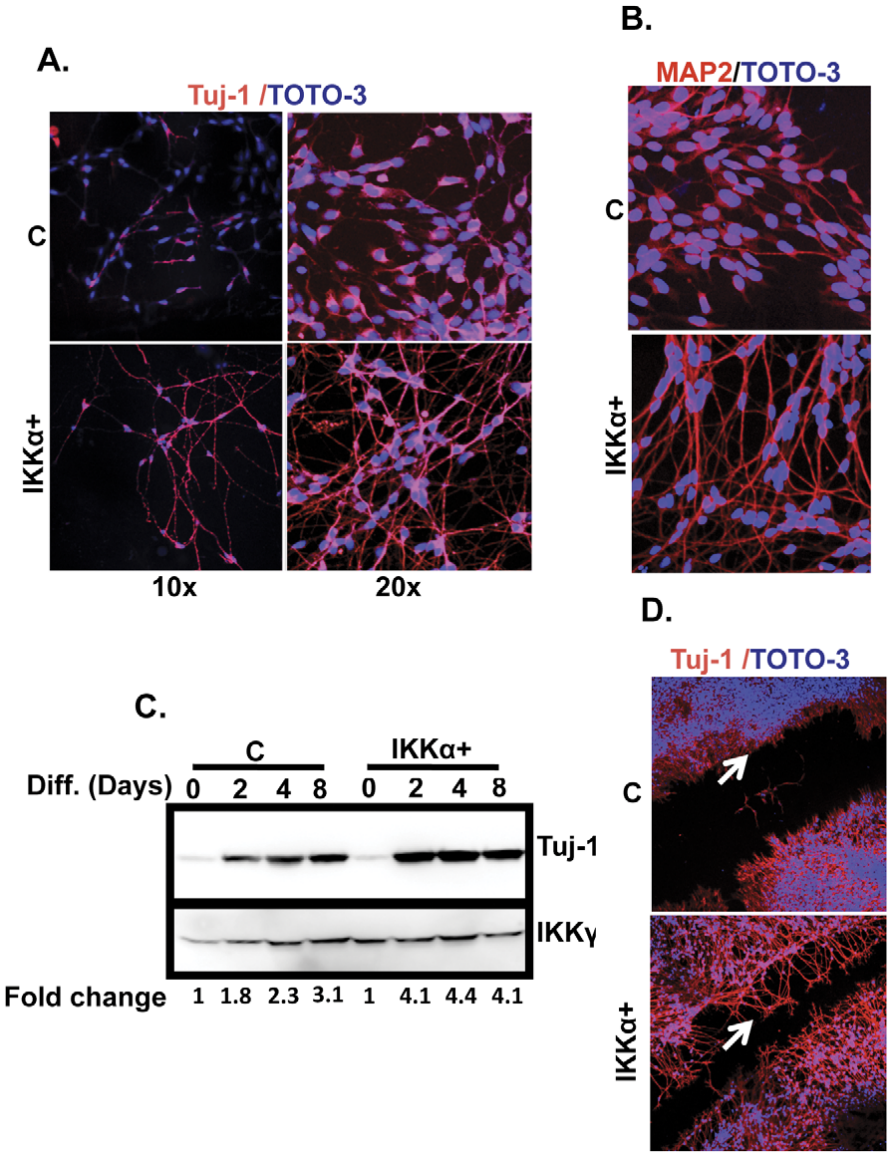
Elevated IKKα promotes the differentiation of MESC2.10 cells. (**A, B**) IKKα promotes neurite outgrowth in differentiating NPCs. Control (C) and IKKα+NPCs were differentiated on coverslips for 4 days, fixed and stained with neuronal differentiation markers Tuj-1 (A) and MAP-2 (B). Representative micrographs obtained with a confocal microscope are shown. The DNA stain TOTO-3 was used to identify nuclei. (**C**) Tuj-1 levels are elevated in differentiating IKKα+ NPCs. Representative western blot results are shown for cytoplasmic lysates staining for Tuj-1 levels at different time points during the differentiation of control and IKKα+ NPCs [diff.(days)]. IKKγ was used as a loading control. Fold-change was obtained by dividing the intensity of Tuj-1 to the corresponding IKKγ, obtained by a Fluorchem 8900 (Alpha Innotech, San Leandro, CA). (**D**) The scratch assay shows extensive neurite outgrowth in differentiating IKKα+ NPCs. Cultures on the 2^nd^ day of differentiation were wounded by a micropipette tip and further incubated for additional two days. Cells were fixed and stained as above. Arrows point to the areas of neurite extension.

To gain more insights in the role of elevated IKKα on NPCs differentiation, control and IKKα+ cells were cultured on laminin-coated dishes and induced to differentiate under conditions that promote the generation of dopaminergic neurons [20]. The majority of cells in differentiating control and IKKα+ are positive for the neuronal markers Tuj-1 and MAP2 by the 4^th^ day. However, ∼50% of the control cells are weakly stained for the expression of Tuj-1 and MAP2 (Fig. 2A, B). Using Western blot analysis, we find that the level of Tuj-1 protein is ∼2.2 fold higher in differentiating IKKα+ NPCs by the 2^nd^ and 4^th^ days compared to controls (Fig. 2C). Moreover, IKKα+ cells display elaborate neurite outgrowth, which is minimal in control MESC2.10 NPCs (Fig. 2A, B). The ability of IKKα to enhance neurite outgrowth was further examined in a scratch lesion assay, which involves removing cells manually and following growth into the open space over time [23]. Differentiating IKKα+ NPCs generate extensive neurite outgrowth two days after the lesion is formed whereas outgrowth is much less in control NPCs (Fig. 2D, arrows). Conditioned medium from differentiating IKKα+ NPCs has no visible effect on the differentiation of the control cells (data not shown), indicating that the affects of IKKα are likely cell autonomous. However, we cannot rule out the possibility of a low level of growth factors or labile molecules secreted by IKKα+ cells that may affect neurite outgrowth. Transient transfection of embryonic rat cortical progenitor cells with IKKα also promotes extensive neurite outgrowth, indicating that the pro-differentiating properties of elevated IKKα are not limited to MESC2.10 human NPCs (Supplementary Fig. 3).

**Figure 3:**
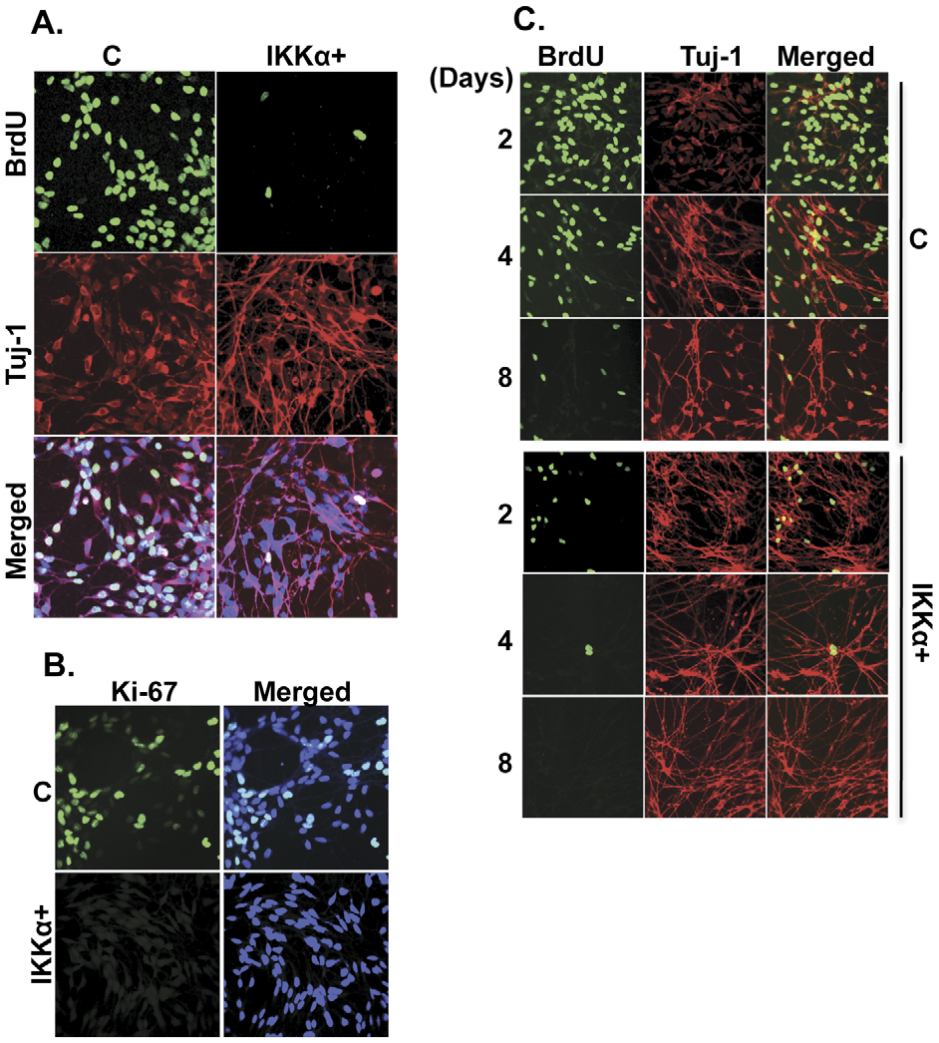
IKKα inhibits the proliferation of early differentiating NPCs. (**A**) Elevated IKKα accelerates NPCs commitment to differentiation. BrdU was added on the 4^th^ day of differentiation. 24 h later cells were fixed and stained with a rat anti-BrdU antibody (green) and the neuron-specific marker Tuj-1. Pictures were taken with a confocal microscope. (**B**) As a further test of proliferation, 4^th^ day cultures were stained with the Ki-67 antibody, which identifies proliferating cells. (**C**) A time-course (days) of BrdU incorporation reveals the difference in rate of decline in proliferation between the control and the IKKα+ NPCs. Each time point represents 24 h of BrdU incorporation. Samples were processed as in A.

While IKKα+ NPCs rapidly cease proliferation upon the induction of differentiation, control cells undergo further divisions as monitored microscopically (data not shown). To examine this phenomenon in detail, we performed BrdU labeling, which is a marker of DNA synthesis and cell proliferation [24]. We find that ∼50% of control NPCs incorporate BrdU at 4 days post-differentiation. However, BrdU incorporation is minimal in differentiating IKKα+ progenitors and ∼90% are post-mitotic (Fig. 3A). Moreover, ∼40% of the 4^th^ day differentiating control NPCs express Ki-67, another marker of cell proliferation [25], whereas less than 1% of IKKα+ cells stain positively for Ki-67 at this time point (Fig. 3B). The majority of BrdU-positive cells stain weakly for Tuj-1, indicating that are not fully committed to differentiation (Figs. 3A, C). However, BrdU incorporation is reduced dramatically upon further incubation and the majority of control NPCs become Tuj-1 positive after 8^th^ day in culture (Fig. 3C). These findings are consistent with those in Figs, 1 and 2, where elevated IKKα blocks the self-renewal of NPCs and promotes the differentiation of MESC2.10 NPCs.

### IKKα affects the REST/NRSF and miR-124 regulatory loop

**Figure 4:**
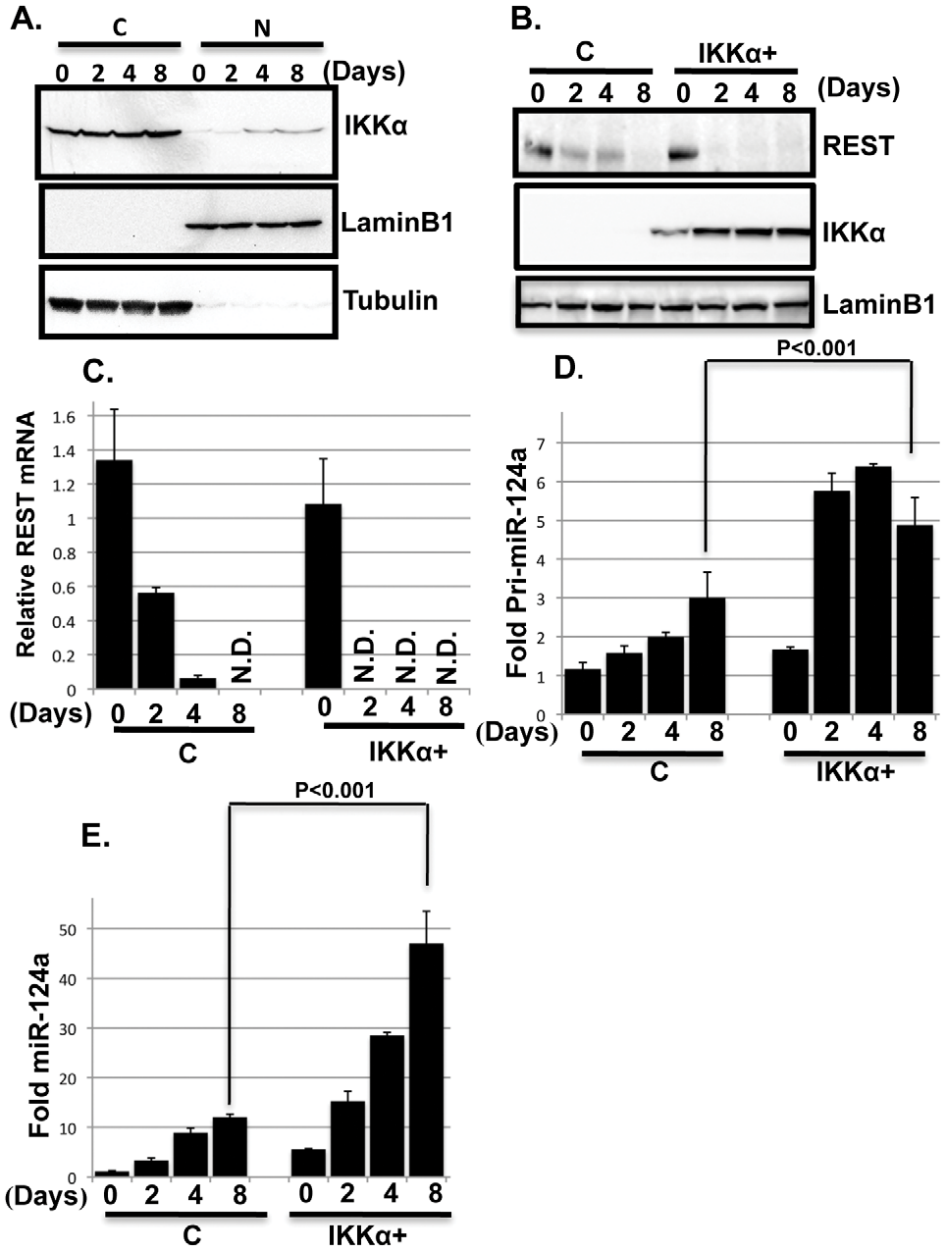
IKKα regulates REST and miR-124a expression. (**A**) IKKα accumulates in the nuclei of differentiating MESC2.10 NPCs. Representative Western blot results for levels of endogenous IKKα in the cytoplasm (C) and nuclear (N) fractions of differentiating NPCs (top panel) are shown. IKKα was detected with a mouse anti-IKKα antibody. Nuclear LaminB1 and cytoplasmic tubulin were used as loading controls (middle and bottom panels, respectively). (**B**) REST protein levels also decline faster in differentiating IKKα+ NPCs compared to differentiating controls. Representative western blot results are shown from nuclear lysates for REST (top panel), IKKα (middle panel) and laminB1 (bottom panel). REST was detected with a mouse anti-REST antibody and Anti-Flag antibody was used to detect IKKα. LaminB1 was used a as loading control. (**C**) After initiating differentiation, REST mRNA levels decline faster in IKKα+ NPCs than in control cells. Taqman probes were used to quantify the mRNA levels at the days shown. The data are shown relative to the level in proliferating control NPCs. GAPDH mRNA was used for normalization. Triplicate samples were averaged for each point, and the SEMs indicated. N.D., not detected. (**D, E**) The accumulation of primary (pri-miRNA) and mature miRNA-124a are shown in D and E, respectively. Taqman probes were used for the qPCR. Pri-miRNA was normalized to GAPDH mRNA and mature miRNA was normalized to the small RNA, RNU6. The data are shown relative to the levels in proliferating control NPCs. P values were obtained using student's t-test.

We examined whether the levels or the cellular distribution of endogenous IKKα is altered during the differentiation of control NPCs. While the levels of IKKα do not change significantly, its accumulation in the nuclear fraction increases in the 4^th^ and 8^th^ day cultures (Fig. 4A). It is relevant that levels of nuclear IKKα increase by the 2^nd^ day in differentiating IKKα+ NPCs (Fig. 4B, middle panel). Thus, the rate of nuclear accumulation of IKKα may contribute to the onset of neuronal differentiation.

KKα is a chromatin modifying kinase and is known to influence gene expression by various means [4], [10], [11]. Since nuclear accumulation of IKKα coincides with neuronal differentiation, we hypothesized that IKKα may directly affect the expression of key regulators of neurogenesis. One prominent modulator of neuronal differentiation is REST/NRSF [26]. REST binds to a consensus cis-element in the promoter of several hundred neuron-specific genes and prevents their expression. The inhibitory functions of REST are essential for the self-renewal of embryonic as well as adult NSCs [27]–[29]. REST levels are dramatically reduced during neuronal differentiation allowing the expression of neurogenic proteins and non-coding RNAs [27], [29]. Using reverse transcription and real-time PCR (qRT-PCR), we find that, while the levels of REST mRNA gradually decrease during the differentiation of control NPCs, it is still detectable by 4 days. In contrast, REST expression is rapidly reduced in differentiating IKKα+ NPCs and is not detectable by the 2^nd^ day post-differentiation (Fig. 4C). Western blot analysis of nuclear lysates is consistent with the mRNA results in differentiating control and IKKα+ NPCs (Fig. 4B, top panel). Thus, compared to control NPCs, the levels of REST mRNA and protein drop more rapidly in differentiating IKKα+ NPCs. REST promoters contain several NF-κB binding sites [30]. Since IKKα regulates NF-κB [4], we hypothesized that it may influence the binding of NF-κB to REST promoter and affect REST expression directly. However, in gene reporter assays with the REST promoter fused to luciferase [30], elevated IKKα does not reduce REST promoter activity (data not shown). Therefore, the effect of IKKα on reduced expression of REST in differentiating IKKα+ NPCs appears to be indirect.

In NPCs REST represses miR-124 expression, which is pro-neurogenic and has several REST binding sites in its promoter [27]. MiR-124 is abundant in neurons and is a major determinant of neuronal differentiation [27], [31]. We hypothesized that IKKα may enhance the expression of miR-124, which may be the underlying cause of REST reduction in differentiating IKKα+ NPCs. MiR-124 has several isoforms and miR-124a is well characterized in the context of neuronal differentiation [27], [31]. We find that miR-124a expression is induced in both control and IKKα+ NPCs during differentiation. However, compared to control, primary as well as the mature miR-124a transcripts are several fold higher in the IKKα+ cells (Fig. 4D, E, respectively). miR-124a levels are inversely related to those of REST in control and IKKα+ NPCs (Fig. 4B, C). Using a gene reporter assay with the miR124a promoter fused to luciferase [27], elevated IKKα does not induce miR-124a promoter activity (data not shown). Taken together, these findings indicate that the levels of REST and miR-124a, which are critical determinants of neuronal differentiation, are significantly altered in IKKα+ NPCs compared to controls. However, our studies do not support a direct link between elevated IKKα and the expression of REST and miR-124.

We further examined differentiating NPCs for expression of other neuron-enriched miRNAs. Interestingly, miR-7, which promotes neurite outgrowth and is co-expressed with miR-124 in other cell models [32], is selectively induced in differentiating IKKα+ NPCs (Fig. S4). The induction of miR-7 may contribute to the extensive neurite outgrowth observed in differentiating IKKα+ NPCs (Figs. 2A, B; Fig. S3). Expression of other miRNAs such as miR-132 and -133a, and -155 is not significantly altered in either differentiating control or IKKα+ NPCs (data not shown).

### Elevated IKKα promotes neuronal maturation

**Figure 5:**
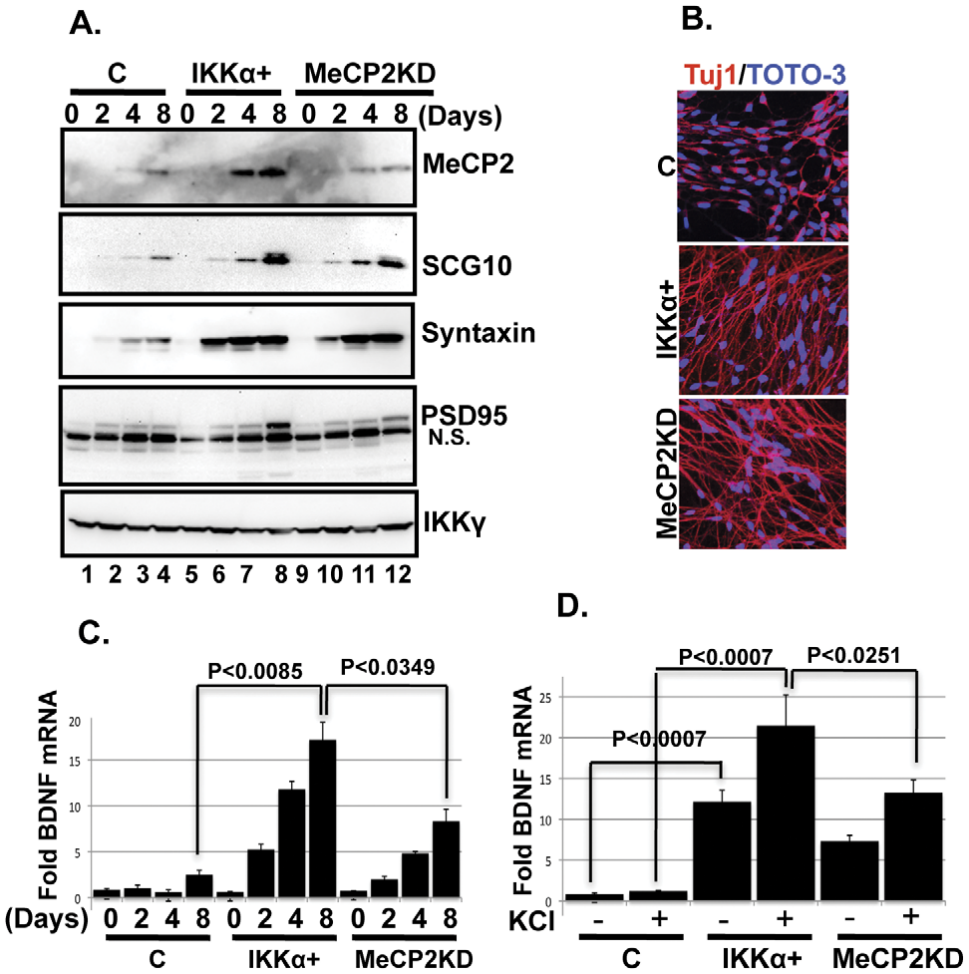
IKKα promotes expression of markers of mature neurons and BDNF. (**A**) Elevated IKKα+ enhances neuronal maturation. Cell lysates from various time points (days after inducing differentiation) were examined by western blotting for the levels MeCP2, SCG10, syntaxin, and PSD-95. A lentivirus encoding an shRNA targeting MeCP2 was used to knockdown the expression of MeCP2 in IKKα+ NPCs (labeled as MeCP2KD) (lanes 9–12). IKKγ was used as a loading control. A non-specific band (N.S., below the authentic band) is recognized by the anti-PSD95 antibody. (**B**) Knockdown of MeCP2 expression does not influence IKKα-induced neuronal differentiation. NPCs were differentiated and examined as described in Fig. 2A. Representative confocal micrographs of 4^th^ day differentiating cultures are shown. (**C**) IKKα promotes MeCP2-dependent BDNF expression. A time course (days) for BDNF expression during the differentiation of NPCs is shown. Taqman probes were used to quantify mRNA generated from the exon-IV of the BDNF promoter. GAPDH was used for normalization. The data are shown relative to the level in proliferating control (day 0) NPCs. (**D**) IKKα also promotes MeCP2-dependent BDNF expression following depolarization. Data are shown for cultures after 8^th^ days of differentiation and depolarization with 50 mM KCl for 6 hr immediately preceding harvest. The data are shown relative to the level in non-depolarized control cells. Assays were done in triplicate and P values were obtained using student's t-test.

**Figure 6:**
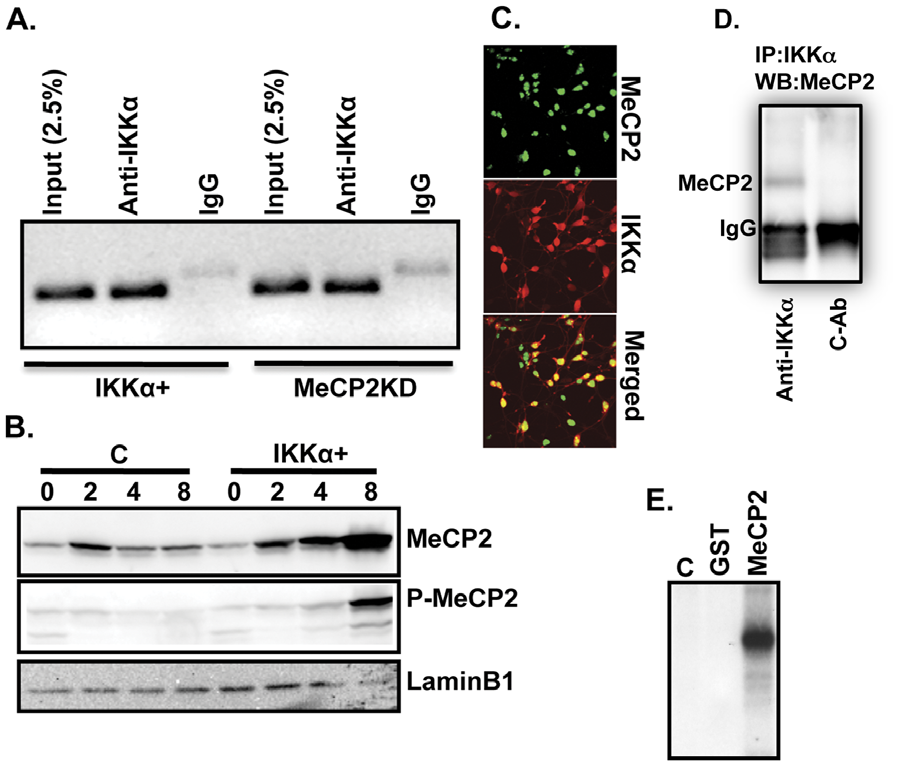
IKKα associates with MeCP2 and is recruited to the exon-IV BDNF promoter. (**A**) Flag-tagged IKKα is recruited to the exon-IV BDNF promoter. ChIP assays were used to immunoprecipitate IKKα/DNA complexes (using anti-Flag antibody) from differentiated IKKα+ and MeCP2KD neurons (day 8). The left panel is ChIP from IKKα+ and the right panel is from MeCP2KD neurons. Non-reactive IgGs were used controls. DNA was amplified by PCR. Products were visualized by agarose gel-electrophoresis and ethidium bromide staining. (**B**) Western blots were used to assay nuclear lysates for phosphorylation of MeCP2 at Ser421 in 8^th^ day differentiated IKKα+ neurons (middle panel). The top panel shows the total levels of MeCP2 during differentiation (0–8 days). LaminB1 was used as a loading control. (**C**) IKKα and MeCP2 co-localize in the nuclei of IKKα+ neurons. IKKα+ NPCs were differentiated for 6 days and stained with MeCP2 antibody (green) and an anti-Flag antibody detecting IKKα (red). Representative micrographs obtained with a confocal microscope are shown. (**D**) MeCP2 co-immunoprecipitates with IKKα. Nuclear lysates from 8^th^ day differentiated IKKα+ neurons were immunoprecipitated with anti-Flag beads (for IKKα) and examined for the presence of MeCP2 with an anti-MeCP2 antibody. A non-immune mouse antibody (C-Ab) was used as a negative control for immunoprecipitation. A strong band for anti-IgG staining is also seen. (**E**) IKKα phosphorylates MeCP2. Active recombinant IKKα was tested for the ability to phosphorylate MeCP2. The kinase assay was performed as described in Methods with recombinant MeCP2 or GST as substrates. Products were visualized by SDS-PAGE followed by autoradiography.

The positive effects of IKKα on neuronal differentiation raised the question of whether it also influences neuronal maturation. One hallmark of maturing neurons is the accumulation of MeCP2, which regulates many aspects of neurodevelopment, and loss of MeCP2 function is implicated in the brain disorder, Rett syndrome [33]. We find that MeCP2 is expressed at a low level in control NPCs, however it is more abundant (∼6 fold higher) in IKKα+ NPCs differentiated for 8 days (Figs. 5A and 6B, top panels). MeCP2 transcription is not altered during the differentiation of control and IKKα+ NPCs, indicating that other post-transcriptional regulatory pathways affect MeCP2 levels (data not shown). Expression of SCG10/Stathmin-2, a neuron-specific microtubule destabilizing protein that promotes neurite outgrowth and neuronal migration [34], and pre-and post-synaptic markers such as syntaxin1 and PSD95 [35], [36] is also induced in IKKα+ cells (Fig. 5A, panels 2–4, lanes 5–8).

Since MeCP2 is important in neurodevelopment [33], we asked whether MeCP2 induction contributes to the differentiation of IKKα+ NPCs. Towards this end, we reduced the expression of MeCP2 in IKKα+ cells using a lentivirus encoding an shRNA targeting MeCP2 (Fig. 5A, top panel, lanes 9–12). This cell line is labeled as MeCP2 knockdown (MeCP2KD). The levels of MeCP2 in the MeCP2KD line are comparable to those detected in differentiating control NPCs (Fig. 5A, top panel; compare lanes 1–4 with 9–12). Knockdown of MeCP2 does not affect neuronal differentiation since the levels of REST and miR-124a are similar to those expressed in IKKα+ cells (data not shown). Moreover, reduction of MeCP2 has no visible effect on IKKα-induced neurite outgrowth, as observed by Tuj-1 staining (Fig. 5B). However, IKKα-induced accumulation of PSD95 in 8^th^ day cultures is significantly reduced (2.4-fold) when MeCP2 expression is silenced (Fig. 5A, panel 4, compare lanes 8 and 12). This is consistent with previous findings in animal models where the absence of functional MeCP2 negatively affects PSD95 levels [37].

### IKKα induces MeCP2-dependent BDNF expression

MeCP2 binds to methylated CpG dinucleotides, which are abundant in the promoters of many genes [38]. MeCP2 is also implicated in the expression of many neuronal genes, including BDNF, whose expression is influenced by the level as well as post-translational modifications of MeCP2 [39], [40]. We asked if elevated MeCP2 in IKKα+ neurons could also affect BDNF levels. BDNF expression can be initiated from 9 different promoters [41] and the exon-IV promoter of human BDNF (rat promoter III) is a well-known target of MeCP2 [39], [40]. Using qRT-PCR, we find that differentiating control NPCs do not express high levels of BDNF (Fig. 5C). However, BDNF transcription from exon-IV is significantly induced in IKKα+ differentiating NPCs and is further elevated by KCl-mediated depolarization of 8^th^ day IKKα+ neurons (Figs. 5C, D). Knockdown of MeCP2 levels reduces both basal as well as KCl-induced BDNF expression by ∼50% (Fig. 5C, D). Thus, IKKα promotes BDNF transcription, which is in part MeCP2-dependent.

IKKα is recruited to several different promoters including NF-κB and estrogen-regulated genes [4], [11]. Since BDNF levels are elevated in IKKα+ neurons, we asked whether IKKα associates with regulatory regions of the exon-IV BDNF promoter. Using chromatin immunoprecipitation (ChIP), we find that IKKα is enriched at the BDNF promoter (Fig. 6A). Moreover, CREB and MeCP2, which bind to this element [42], [43], are also abundant (Fig. S5A, B, left panels). As expected, MeCP2 binding to the exon-IV BDNF promoter is reduced in MeCP2KD neurons (Fig. S5B, right panel), which coincides with the reduction of BDNF expression (Fig. 5C, D) and suggests that the concentration of MeCP2 may be important for the regulation of the exon-IV BDNF promoter. Interestingly, the association of IKKα and CREB with the BDNF promoter is not altered by knockdown of MeCP2 levels, indicating that they may bind independently of MeCP2 (Figs. 6A and S5A, right panels). However, we cannot rule out the possibility that residual MeCP2, which is bound to the exon-IV promoter in MeCP2KD neurons, may be sufficient to recruit IKKα and CREB (Fig. S5B, right panel). Overall, these findings support a role for IKKα in the regulation of MeCP2-dependent BDNF expression.

Phosphorylation of MeCP2 at Ser421 has previously been implicated in the induction of BDNF expression [39]. Using an antibody recognizing phospho-Ser 421, we find that phosphorylated MeCP2 accumulates in 8^th^ day differentiated IKKα+ but not control neurons (Fig. 6B, middle panel). This time course coincides with the elevated levels of BDNF in IKKα+ neurons (Fig. 5C, D). The fact that IKKα is a kinase raised the question of whether IKKα associates with and phosphorylates MeCP2. IKKα co-localizes with MeCP2 in the nuclei of IKKα+ neurons (Fig. 6C). Moreover, complexes containing both IKKα and MeCP2 can be immunoprecipitated from the nuclear fraction of 8^th^ day post-differentiation IKKα+ neurons (Fig. 6B, D). Therefore, we performed *in vitro* kinase assays using recombinant IKKα and MeCP2 proteins. We find that IKKα phosphorylates MeCP2 (Fig. 6E). However, mass spectrometric analysis identifies phosphorylated Ser residues other than Ser421 (A. Khoshnan, et al., unpublished data). Previous studies have identified CAMK-II and CAMK-IV as potential kinases phosphorylating Ser421 of MeCP2 [39], [44]. Thus, phosphorylation of Ser421 in IKKα+ neurons may be an indirect effect of IKKα. The characterization of IKKα-mediated phosphorylation of MeCP2 at Ser421 and other residues and their effects on the activity of MeCP2 is a topic of current work in our laboratory.

## Discussion

We have identified novel functions for IKKα in enhancing the differentiation of human NPCs. Elevated IKKα indirectly lowers the level of REST/NRSF repressor, which is a global inhibitor of neurogenesis [26]–[29]. The ability of IKKα to enhance neuronal differentiation is further exemplified by the induction of neuron-enriched miRNAs such as miR-124a and -7, and proteins including MeCP2, PSD95, and BDNF, which are involved in neurite outgrowth, neuronal maturation, and synaptic plasticity. Thus, increasing the level and/or the activity of IKKα may be a useful strategy to promote neuronal differentiation *in vitro* and potentially *in vivo*. Our results also highlight a direct link between IKKα and MeCP2, which could be instrumental in regulating MeCP2-dependent gene expression and neurodevelopment.

Elevation of IKKα inhibits self-renewal and accelerates the differentiation of MESC2.10 NPCs, and reduction of REST expression may play a role. As a repressor of neuronal genes, REST promotes the proliferation of NSCs as well as neuroblastoma cell lines, whereas reduction of REST induces neuronal differentiation [26]–[29], [45]. We propose that the effect of IKKα on REST expression is indirect, since elevated IKKα does not lower the REST promoter activity. However, REST promoters have several NF-κB binding sites [30] and the regulation of NF-κB activity by IKKα may influence REST levels under certain physiological conditions. We have not been successful in establishing a link between IKKα/NF-κB and REST transcription, however.

A critical step in the initiation of NSC differentiation is the induction of miR-124, which is repressed by REST [27]. miR-124 is enriched in the brain and is recognized as the “micromanager of neurogenesis” *in vivo* [46], [47]. Indeed, miR-124 promotes the direct conversion of human fibroblasts into functional neurons, where it instructs chromatin remodeling and promotes brain-specific alternative splicing of mRNAs essential for neuronal differentiation [48]–[50]. Thus, the reduced levels of REST and reciprocal elevation of miR-124 in IKKα+ cells will likely cause global changes in gene expression that inhibit proliferation and engage the differentiation programming (Fig. 4). In addition, miR-124 plays an important role in synaptic plasticity and memory formation in post-mitotic neurons in Aplysia [51]. *In vivo* studies indicate that IKKα is involved in hippocampal-dependent memory reconsolidation [10]. It will be interesting to examine whether elevated expression of IKKα induces miR-124 and enhances memory formation and learning, possibly by affecting neurogenesis in the adult hippocampus.

IKKα accumulates in the nuclei of differentiating NPCs (Figs. 4A, B, and 6C), and nuclear transfer of IKKα is implicated in the phosphorylation of histone-3 (Ser10), which leads to enhanced expression of various genes [4], [10]. Our transcriptome analysis (mRNA–seq) of differentiating control and IKKα+ NPCs reveals significant changes in the expression of several hundred mRNAs in IKKα+ cells; some of these encode proteins involved in neurodevelopment and the splicing of neuron-specific mRNAs (A. Khoshnan et al., unpublished data). Characterization of some of these genes may shed further light on the mechanism of how IKKα accelerates neuronal differentiation and regulates complex epigenetic changes such as neurite outgrowth. It is intriguing that miR-7, which is implicated in neuronal homeostasis and neurite outgrowth [32], is selectively induced in differentiating IKKα+ NPCs. miR-7 also protects dopaminergic neurons against oxidative stress, where it reduces the expression of α-synuclein and leads to enhanced survival [52]. We have previously shown that IKKα protects MESC2.10 neurons against oxidative stress-induced neuronal death and preserves the integrity of neuron-enriched huntingtin protein, which has neuroprotective properties [13]. Thus, in addition to promoting neurite outgrowth, IKKα-induced miR-7 may also contribute to the resiliency of neurons under adverse environmental conditions.

The ability of IKKα to regulate MeCP2 levels and activity is another novel aspect of this study. These interactions were characterized in the context of BDNF expression, which is induced by elevated IKKα and suppressed when MeCP2 levels are knocked down (Fig. 5). BDNF plays a critical role in neuronal differentiation and survival, miRNA processing, and synaptic plasticity [53], [54]. The MeCP2-dependent induction of BDNF may therefore be important in these processes, which has implications for neurological and psychiatric disorders. While earlier studies supported an inhibitory role for MeCP2, recent findings are consistent with a positive effect of MeCP2 on BDNF expression [39], [40], [42], [43]. Moreover, in animal models where MeCP2 is inactive or deleted, BDNF levels are significantly reduced [55], [56]. Our data are also consistent with a positive effect of elevated MeCP2 on BDNF and highlight the involvement of IKKα.

Recent studies propose that MeCP2 may function both as a repressor and activator of the same target genes, depending on its association with other proteins. For example, MeCP2-dependent recruitment of HDAC2 or CREB to the glial-derived neurotrophic factor promoter can inhibit or promote gene expression, respectively [57]. We find that IKKα associates with MeCP2 and both are recruited to the BDNF exon-IV promoter, which may be crucial for the induction of BDNF. Thus, similar to CREB, binding of IKKα to MeCP2 may enhance MeCP2-dependent gene expression. Moreover, maximal BDNF expression in IKKα+ neurons coincides with elevated levels of MeCP2 (Fig. 5). We posit that changes in the homeostasis of MeCP2 may dictate whether it acts as repressor or activator of gene expression. At steady state, MeCP2 may simply function as a chromatin organizer and control the noise in global gene expression [38]. On the other hand, when MeCP2 levels are elevated, it may facilitate selective gene expression by associating by other regulatory proteins such as IKKα and CREB. It is relevant that elevation of MeCP2 in transgenic mice induces the expression of ∼2200 genes including CREB [14]. Moreover, the levels of MeCP2 and its phosphorylation at Ser421 are increased by exogenous factors such as amphetamine, cocaine, and the anti-depressant fluoxetine [58], [59]. These findings support the dynamic nature of MeCP2 expression in neurons and how fluctuations in its levels and/or its phosphorylation may dictate various functions. Exogenous stimuli including growth factors and cytokines also regulate IKKα activity [1]–[4]. The elevation of MeCP2 in IKKα+ neurons and the phosphorylation of MeCP2 by IKKα raise the possibility that environmental activation of IKKα may affect MeCP2 homeostasis and activity. Further characterization of IKKα-MeCP2 interactions may shed light on the complex nature of MeCP2 activities in neurons.

## Materials and Methods

### Antibodies and reagents

Anti-Tuj-1 antibody was obtained from Covance (Berkeley, CA). Anti-Nestin antibody was purchased from R&D systems (Minneapolis, MN). Anti-syntaxin and anti-Flag antibodies were obtained from Sigma (St Luis, MO). Anti-NRSF/REST and anti-PSD95 antibodies were provided by David Anderson and Mary Kennedy at the California Institute of Technology, respectively. Anti-IKKα was purchased from BD Biosciences (San Diego, CA). Anti-MeCP2 antibody and recombinant active IKKα were purchased from Millipore (Temecula, CA). Anti-phospho MeCP2 (Ser421) antibody was provided by Dr. Michael Greenberg at Harvard medical school. Anti- IKKγ and anti-MAP2 antibodies were obtained from Cell Signaling Technology (Danvers, MA). Anti-SCG10 antibody was produced in house. Rat anti-BrdU and ki67 rabbit antibodies were purchased from abcam (Cambridge, MA). Anti-laminB1 antibody, DMEM/F12, bFGF-2, N-2 and B-27 media supplements were obtained from Invitrogen (Carlsbad, CA). Anti-CREB antibody was purchased from Santa Cruz Biotechnology (Santa Cruz, CA). Cell fractionation and ECL detection kits including HRP-conjugated secondary antibodies were from PIERCE Biotechnology (Rockford, IL). Recombinant MeCP2 protein was purchased from Panomic (Santa Clara, CA).

### Generation of MESC2.10 human neurons

The generation of MESC2.10 human NPCs has previously been reported [20]. Briefly, NPCs were obtained from an 8 week-old human embryo and transduced with a retrovirus encoding a tetracycline-regulated (tet-off) v-myc to promote proliferation [20]. Propagation is in serum-free medium containing bFGF-2 [13], [20]. In our studies, NPCs were propagated in dishes coated with poly-lysine and laminin in DMEM/F12 in the presence N2 and B-27 neuronal supplements and 20 ng/ml bFGF-2 (Invitrogen). When indicated, doxycycline (2 µg/ml) was added to proliferating medium to block the expression of v-myc. To differentiate MESC2.10 NPCs, proliferation medium was replaced DMEM/F12 containing N2 supplement, 2 µg/ml of doxycycline and 20 µM cAMP.

### Neurosphere assays

Single-cell suspensions of MESC2.10 and IKKα+ NPCs (5,000 cells/ml) were cultured in DMEM/F12, containing bFGF (40 ng/ml) and B27 supplements in the presence of 2 µg/ml of doxycycline for 6 days. For secondary neurosphere assays, primary neurospheres were dissociated using trypL (Invitrogen) and cultured as above. Fresh medium containing FGF-2 and doxycycline was added every two days. Cultures were examined using a phase contrast microscope. The number of spheres in 6 wells were counted and averaged for each condition. To stain neurospheres for markers of differentiation, 6 days old cultures (after the first round) were dissociated and plated on laminin substrates for 24 h as above.

### Production of rat cortical NPCs

Procedures for animal work were approved by the California Institute of Technology Institutional Animal Care and Use Committee (protocol #1200). Embryonic day 10 cortical progenitor cells were generated by mincing the brain into small pieces followed by papain dissociation (Worthington Biomedical Corporation, NJ). Cells were cultivated as neurospheres in stem cell medium (DMEM/F12 plus B-27 and N-2 supplement, Invitrogen) in the presence of FGF and EGF (20 ng/ml).

### Immunohistochemisty

Differentiating NPCs were cultured on cover slips and fixed in 4% paraformaldehyde followed by permeabilization in 70% methanol and stained with the antibodies indicated. Secondary antibodies conjugated to FITC or Alexa 568 were used to detect the respective primary antibodies. Pictures were taken with a confocal microscope.

### BrdU Labeling

Differentiating NPCs grown on coverslips were treated with BrdU (1 mM) for 24 hrs. Cells were fixed in 4% paraformaldehyde followed by permeabilization in 70% methanol in PBS at −20°C overnight. To denature chromatin, coverslips were immersed in 2 N HCl for 30 min at 37°C and neutralized in 0.1 M borate buffer pH 8.5 by washing 2 times for 5 min each. To detect BrdU incorporation, coverslips were incubated with Rat-anti-BrdU (1∶200). Anti-Tuj-1 (1∶1000) was used to stain neurons. Goat anti-rat antibody conjugated to Alexa 568 and goat anti-mouse antibody conjugated to FITC (1∶500) were used as secondary antibodies. Pictures were taken with a confocal microscope.

### RNA and miRNA extraction and quantifications

Total RNA was extracted by TriZol and was further purified by RNA purification columns (Qiagen, Valencia, CA). For cDNA synthesis, 250 ng of RNA was reverse transcribed with superscript VILO cDNA synthesis kit (Invitrogen). To quantify REST, primary miR-124a, and BNDF mRNAs, Taqman probes synthesized by Applied Biosystems (Foster City, CA), were used for real-time PCR using a 7300 real-time PCR system (Applied Biosystems). miRNAs were enriched by the mirVana kit (Ambion). Taqman probes from Applied Biosystems were also used to quantify different miRNAs according to provided instructions. Data were analyzed comparatively by the formula 2^(−ΔΔct)^. GAPDH was used for the normalization of different mRNAs, and U6 small nuclear RNA (RNU6) was used for the normalization of miRNAs. Each sample was compared to the mRNA or miRNA levels of proliferating control cells (day 0). Results are shown as fold-changes. Student's t-test was used to calculate the P values.

### Lentiviral production and Western blot analysis

These procedures were performed as described previously [13]. ShRNAs targeting MeCP2 were obtained from Thermo Scientific (Lafayette, CO).

### ChIP assays

For chromatin immunoprecipitations, we used the Magna kit from Millipore (Bedford, MA) and followed the manufacturer's instructions with minor modifications. To fragment the DNA, the following settings on a Misonix 3000 (Misonex, Farmingdale, NY) were used; 12 cycles of 30 sec sonication, 60 sec rest, at a power level of 6 watts followed by 12 cycles of 30 sec sonication/60 sec rest at a power level of 9 watts. Samples were held in ethanol-ice bath to prevent overheating. DNA protein complexes were immunoprecipitated with the indicated antibodies and DNA was cleaned according the procedures provided in the Magna kit. Standard PCR procedures were used to amplify the exon-IV BDNF promoter using the following primers (Forward 5′-ATATGACAGCGCACGTCAAG-3′ and reverse 5′-TCACGTTCCCTTCGCTTAAT-3′). Primers were designed according to the sequence published by Fang et al., [60]. PCR products were examined by agarose gel-electrophoresis and ethidium bromide staining.

### Kinase assay

0.25 µg of active recombinant IKKα was incubated with 0.25 µg of full-length MeCP2 in a kinase buffer in the presence of ^32^P-γ-ATP for 30 min at 30°C [61]. GST protein was used as a negative control. Kinase products were examined by SDS-PAGE and autoradiography.

## Supporting Information

Figure S1 **Western blot analysis of IKKα levels in the control and IKKα expressing (IKKα+) line.** MESC2.10 NPCs were transduced with a recombinant lentivirus encoding a Flag-tagged IKKα as described previously [13]. Quantification reveals that the levels of IKKα in the transduced cells are ∼ three folds higher than that in the control cells.
(TIF)

Figure S2 **Nestin is expressed in proliferating and dissociated day 6 neurospheres.** (**A**) Control and IKKα+ NPCs express Nestin. Cells were plated on laminin and cultivated in proliferating medium for 24 hr. Cells were fixed and stained for Nestin. (**B**) Day 6 neurospheres were dissociated and plated on laminin and cultivated in proliferating medium in the presence of doxycycline for an additional 24 hr. Cells were fixed and stained as in A. Nestin accumulates in the neurites of IKKα+ cells.
(TIF)

Figure S3 **IKKα promotes neurite outgrowth in rat cortical progenitor cells.** Embryonic day 10 cortical progenitor cells were dissociated by mincing the brain into small pieces followed by papain dissociation (Worthington Biomedical Corporation, NJ). Cells were cultivated as neurospheres in stem cell medium (DMEM/F12 plus B-27 and N-2 supplement, Invitrogen) in the presence of FGF and EGF (20 ng/ml). Neurospheres were dissociated with trypsin and cultured on laminin-coated dishes. Using lipofectamine, cells were transfected with empty vector (C/EGFP) or IKKα+EGFP. On the following day, medium without FGF or EGF, containing cAMP (50 nM/ml) was added to promote neuronal differentiation. Cells were examined and pictures taken 4 days post-differentiation. Representative micrographs showing extensive neurite outgrowth in differentiating neurons expressing IKKα are shown. More than 80% of EGFP positive cells in the IKKα expressing cells displayed extensive neurite outgrowth.
(TIF)

Figure S4 **Mature miR-7 accumulates in IKKα+ NPCs.** Small RNAs from differentiating NPCs were obtained as described in Methods. Taqman probes were used for qRT-PCR of mature miR-7. Samples were normalized to the small RNA, RNU6. Each sample was compared to time zero (day 0) of the control (C) proliferating NPCs. Results are shown as fold-change. P values were obtained using the student's t-test.
(TIF)

Figure S5 **CREB and MeCP2 are recruited to the exon-IV BDNF promoter.** ChIP assays using the indicated antibodies were used to immunoprecipitate protein/DNA complexes. The left part of each panel is ChIP from IKKα+ NPCs and the right part of each panel is from MeCP2KD cells. CREB recruitment is shown in (**A**), MeCP2 recruitment is shown in (**B**). Non-reactive IgGs were used a controls. DNA was amplified by PCR. Products were visualized by agarose gel- electrophoresis and ethidium bromide staining.
(TIF)
