# Supplementary figures and images for: Elevated IKKα Accelerates the Differentiation of Human Neuronal Progenitor Cells and Induces MeCP2-Dependent BDNF Expression
Source: PLoS One. 2012 Jul 27;7(7):e41794. doi: 10.1371/journal.pone.0041794 (PMC3407048; doi:10.1371/journal.pone.0041794)

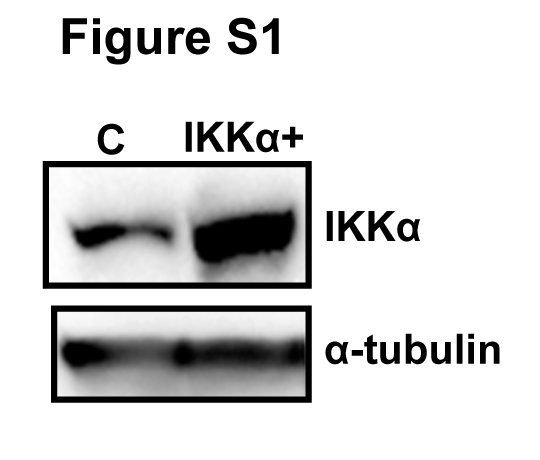

Supplement: Figure S1 — Western blot analysis of IKKα levels in the control and IKKα expressing (IKKα+) line. MESC2.10 NPCs were transduced with a recombinant lentivirus encoding a Flag-tagged IKKα as described previously [13]. Quantification reveals that the levels of IKKα in the transduced cells are ∼ three folds higher than that in the control cells. (TIF) [file pone.0041794.s001.tif]

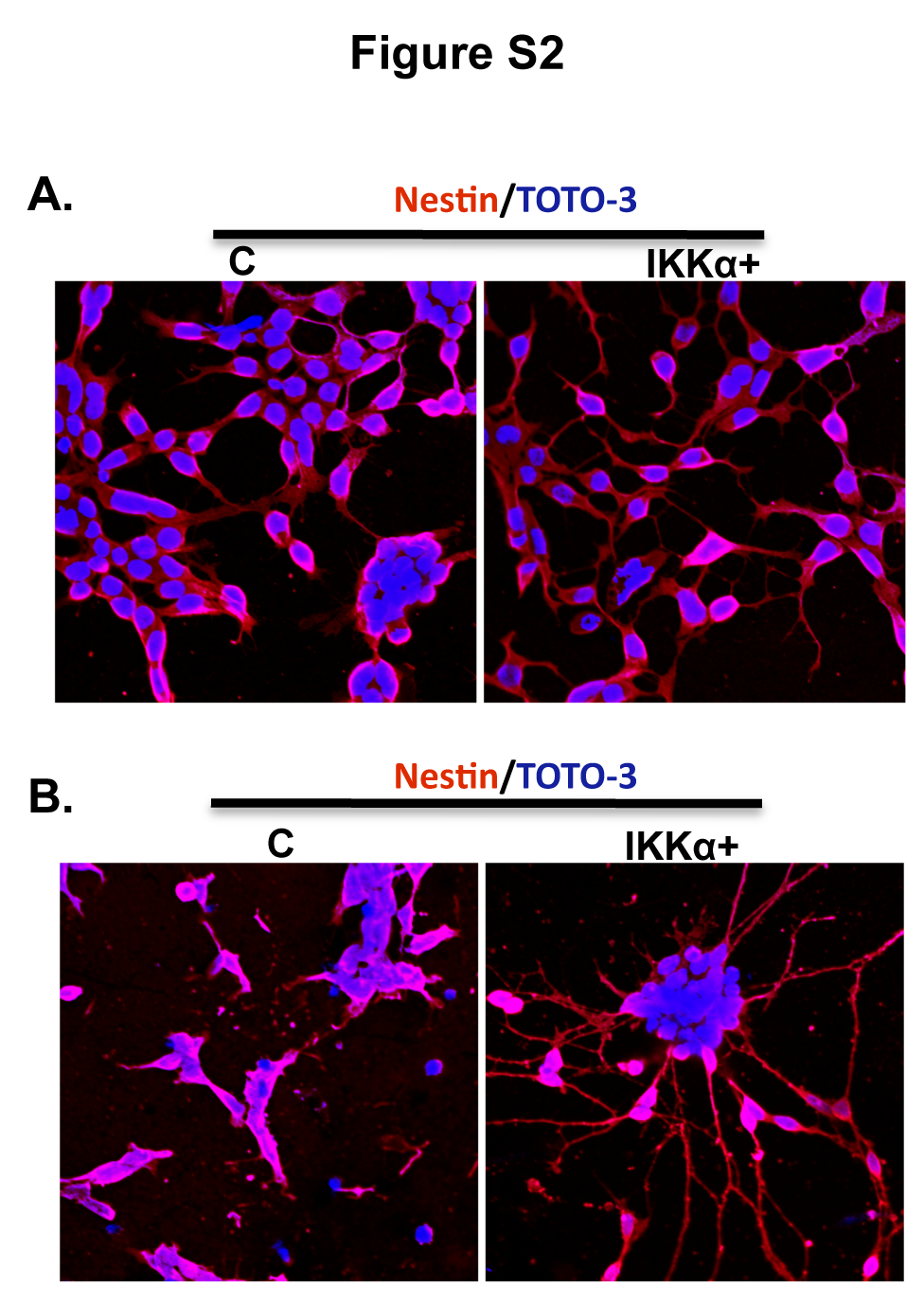

Supplement: Figure S2 — Nestin is expressed in proliferating and dissociated day 6 neurospheres. (A) Control and IKKα+ NPCs express Nestin. Cells were plated on laminin and cultivated in proliferating medium for 24 hr. Cells were fixed and stained for Nestin. (B) Day 6 neurospheres were dissociated and plated on laminin and cultivated in proliferating medium in the presence of doxycycline for an additional 24 hr. Cells were fixed and stained as in A. Nestin accumulates in the neurites of IKKα+ cells. (TIF) [file pone.0041794.s002.tif]

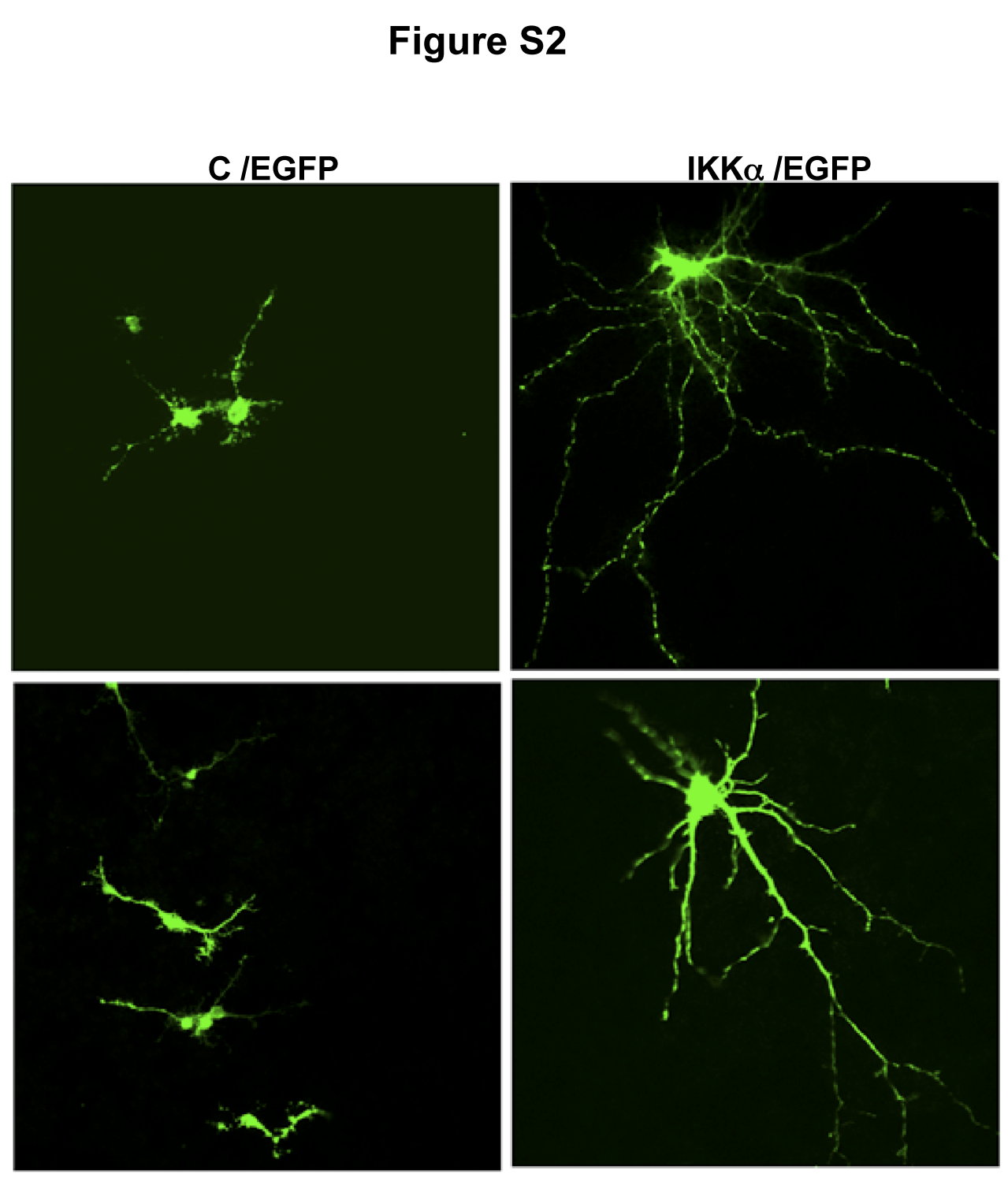

Supplement: Figure S3 — IKKα promotes neurite outgrowth in rat cortical progenitor cells. Embryonic day 10 cortical progenitor cells were dissociated by mincing the brain into small pieces followed by papain dissociation (Worthington Biomedical Corporation, NJ). Cells were cultivated as neurospheres in stem cell medium (DMEM/F12 plus B-27 and N-2 supplement, Invitrogen) in the presence of FGF and EGF (20 ng/ml). Neurospheres were dissociated with trypsin and cultured on laminin-coated dishes. Using lipofectamine, cells were transfected with empty vector (C/EGFP) or IKKα+EGFP. On the following day, medium without FGF or EGF, containing cAMP (50 nM/ml) was added to promote neuronal differentiation. Cells were examined and pictures taken 4 days post-differentiation. Representative micrographs showing extensive neurite outgrowth in differentiating neurons expressing IKKα are shown. More than 80% of EGFP positive cells in the IKKα expressing cells displayed extensive neurite outgrowth. (TIF) [file pone.0041794.s003.tif]

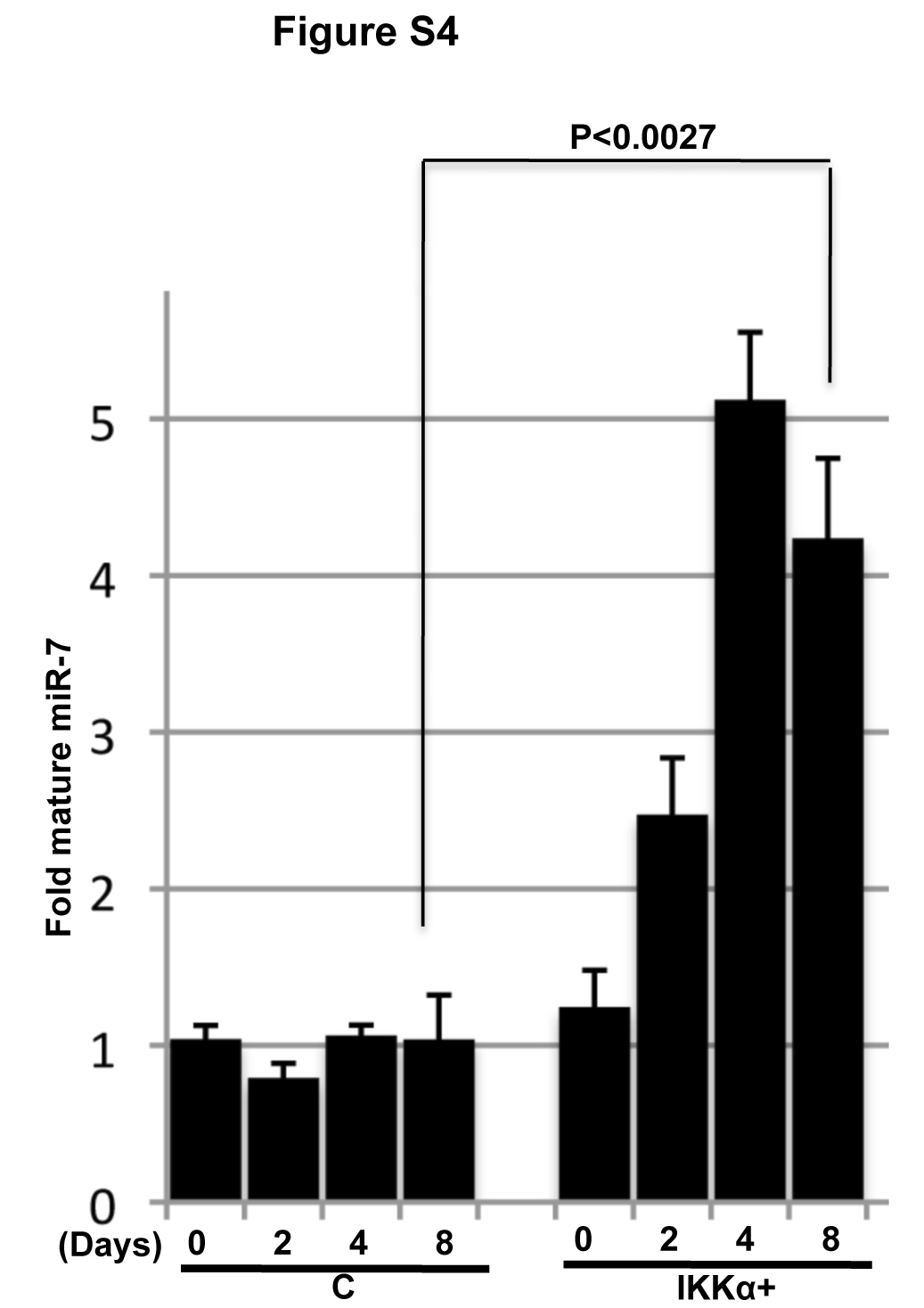

Supplement: Figure S4 — Mature miR-7 accumulates in IKKα+ NPCs. Small RNAs from differentiating NPCs were obtained as described in Methods. Taqman probes were used for qRT-PCR of mature miR-7. Samples were normalized to the small RNA, RNU6. Each sample was compared to time zero (day 0) of the control (C) proliferating NPCs. Results are shown as fold-change. P values were obtained using the student's t-test. (TIF) [file pone.0041794.s004.tif]

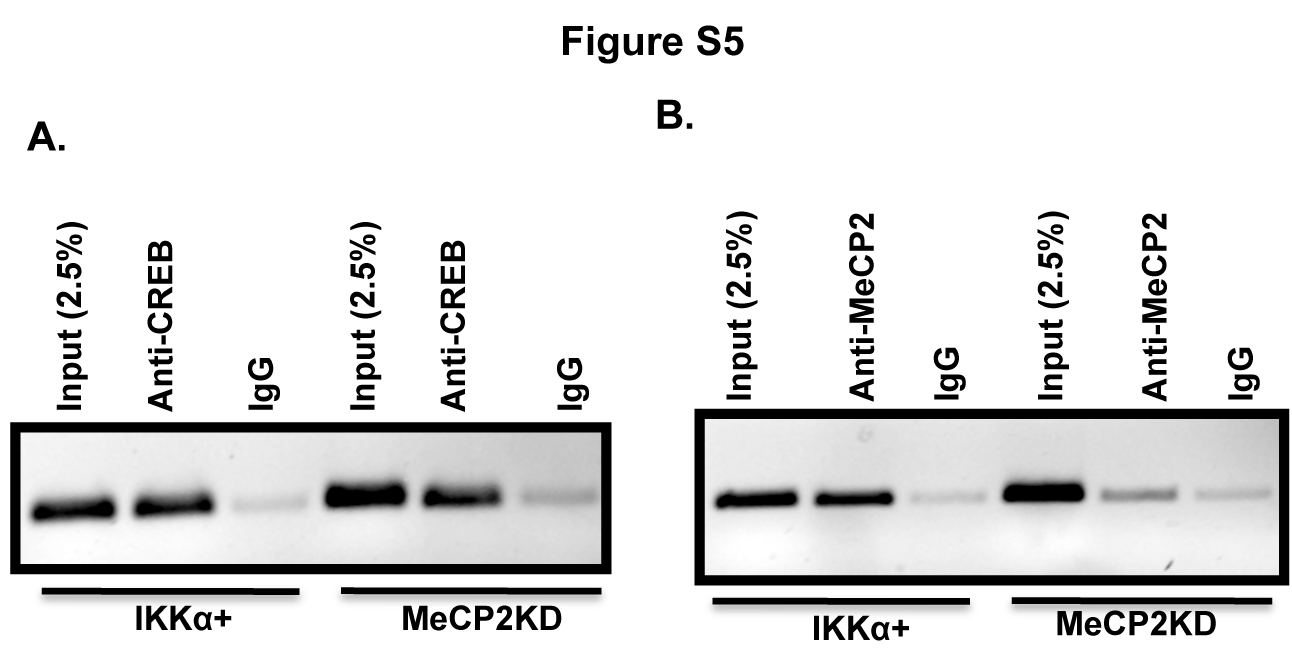

Supplement: Figure S5 — CREB and MeCP2 are recruited to the exon-IV BDNF promoter. ChIP assays using the indicated antibodies were used to immunoprecipitate protein/DNA complexes. The left part of each panel is ChIP from IKKα+ NPCs and the right part of each panel is from MeCP2KD cells. CREB recruitment is shown in (A), MeCP2 recruitment is shown in (B). Non-reactive IgGs were used a controls. DNA was amplified by PCR. Products were visualized by agarose gel- electrophoresis and ethidium bromide staining. (TIF) [file pone.0041794.s005.tif]
